# The extracellular matrix alteration, implication in modulation of drug resistance mechanism: friends or foes?

**DOI:** 10.1186/s13046-022-02484-1

**Published:** 2022-09-16

**Authors:** Ancuta Jurj, Calin Ionescu, Ioana Berindan-Neagoe, Cornelia Braicu

**Affiliations:** 1 Research Center for Functional Genomics, Biomedicine and Translational Medicine, Iuliu Hațieganu University of Medicine and Pharmacy, 400337 Cluj-Napoca, Romania; 2 7Th Surgical Department, Iuliu Hațieganu University of Medicine and Pharmacy, 8 Victor Babes Street, 400012 Cluj-Napoca, Romania; 3 Surgical Department, Municipal Hospital, 400139 Cluj-Napoca, Romania; 4 Research Center for Oncopathology and Translational Medicine (CCOMT), George Emil Palade University of Medicine, Pharmacy, Sciences and Technology, 540139 Targu Mures, Romania

**Keywords:** Cancer, Extracellular matrix, Tumour microenvironment, Drug resistance

## Abstract

The extracellular matrix (ECM) is an important component of the tumor microenvironment (TME), having several important roles related to the hallmarks of cancer. In cancer, multiple components of the ECM have been shown to be altered. Although most of these alterations are represented by the increased or decreased quantity of the ECM components, changes regarding the functional alteration of a particular ECM component or of the ECM as a whole have been described. These alterations can be induced by the cancer cells directly or by the TME cells, with cancer-associated fibroblasts being of particular interest in this regard. Because the ECM has this wide array of functions in the tumor, preclinical and clinical studies have assessed the possibility of targeting the ECM, with some of them showing encouraging results. In the present review, we will highlight the most relevant ECM components presenting a comprehensive description of their physical, cellular and molecular properties which can alter the therapy response of the tumor cells. Lastly, some evidences regarding important biological processes were discussed, offering a more detailed understanding of how to modulate altered signalling pathways and to counteract drug resistance mechanisms in tumor cells.

**Supplementary Information:**

The online version contains supplementary material available at 10.1186/s13046-022-02484-1.

## Introduction

The tumor microenvironment (TME) is composed of the extracellular matrix (ECM) and various stromal cell types, including endothelial cells, immune cells, fibroblasts, and adipocytes. Added to this, the cells of the TME interact via soluble factors, intercellular receptor-ligand interactions, and exosomes [1–3].

The ECM is composed of approximatively 300 unique matrix macromolecules which are classified into collagens, glycoproteins (laminins, elastin, tenascins and fibronectin) and glycoproteins (heparan sulphate proteoglycans, hyaluronan and versican) [4]. Growth factors as well as ECM-associated proteins, interact with the ECM components, making the ECM to have a key role in regulating intercellular communication [5]. Added to this, the ECM from different tissues exhibit different chemical, mechanical and topographical properties, features which critically influence cell fate and function through different mechanosignalling routes [6, 7]. In this review, we discuss about the importance of ECM and its bidirectional interaction with tumor cells and TME constituents in regulating tumor progression and therapy response.

According to its function, composition and location, the ECM can be divided into two main categories: the interstitial matrix and the basement membrane (Table 1). The interstitial matrix forms a three-dimensional network surrounding the malignant clone interconnecting it with various stromal cells. This matrix assures the structural integrity of tissues and organs and modulates biological processes like cell differentiation and migration. In its composition, the interstitial matrix is specifically composed of collagen, fibronectin and elastin, which varies between tissues [8]. In addition, it is known that, in cancer biology, the remodelling of the interstitial ECM induces several critical changes that affect cell signalling, cell migration and tumor progression [9]. The basement membrane presents as a sheet-like dense structure. This membrane consists of collagens and laminins, being interconnected through several proteins such as heparan sulphate, proteoglycans (HSPGs) and nidogen [10]. In the context of cancer the basement membrane is critical in preventing the initial in situ carcinoma of invading the adjacent tissues [11].

**Table 1:** The components of basement membrane and interstitial matrices, and their implication in ECM

Matrix	ECM component	Interact with:	Cell surface receptors	References
Basement membrane	Collagen IV	Laminin, Nidogen, Perlecan, proteoglycans and growth factors (TGFβ1, PDGF)	Integrins and DDR1	[12, 13]
	Laminins	Collagen, laminins, nidogens and perlecan, plasminogen, agrin, sulfatides, immunoglobulins and plectin, plasminogen activator and axon guidance molecules	Cell surface collagens (collagen XVII), dystroglycan, integrins and syndecans	[13, 14]
	Perlecan	Growth factors (FGF, bFGF, VEGF), collagen IV, antithrombin III, sulfatides, laminins, nidogens, fibulins, fibronectin, thrombospondins and prolargin	Integrins	[15, 16]
	Nidogen/ entactin	Collagen IV, laminins, fibulin 1 and perlecan	Integrins	[13, 17]
Interstitial matrices	Collagen I	Cytokines (IL2, oncostatin M), growth factors (PDGF, KGF), collagens, fibronectin, proteoglycans	Syndecans, integrins, DDR1 and DDR2	[18]
	Elastin	Fibrillin, fibulins, elastins, EBP	Integrin Vβ3	[19]
	Fibronectin	Growth factors (PDGF, VEGF, FGF), phospholipids, gangliosides, acetylcholinesterase, factor XIIIa transglutaminase, fibrinogen, fibronectin, fibrin, collagens, fibulin-1, proteoglycans, thrombospondins, factor VIII	Syndecans and integrins	[20, 21]
	Proteoglycans	Growth factors (TGFβ, FGF, VEGF), morphogens (Wnt, BMP), cytokines (CCL2), fibronectin, collagens, laminins, tenascins	Growth factor receptors (VEGFR), L1CAM, integrins	[22]
	Tenascins	Growth factors (VEGF), cytokines (IL8), collagen, fibronectin, proteoglycans	Growth factors receptors (EGFR), integrins, cell surface annexin II	[23]

*DDR1* Discoidin domain-containing receptor 1, *DDR2* Discoidin domain-containing receptor 2, *TGFβ* Transforming growth factor β, *PDGF* Platelet-derived growth factor, *FGF* Fibroblast growth factor, *bFGF* Basic fibroblast growth factor, *VEGF* Vascular endothelial growth factor, *VEGFR* Vascular endothelial growth factor receptor, *L1CAM* L1 cell adhesion molecule, *KGF* Keratinocyte growth factor, *IL2* Interleukin-2, *EBP* Elastin-binding protein, *CCL2* C–C motif chemokine ligand 2, *BMP* Bone morphogenetic protein, *EGFR* Epidermal growth factor receptor

**Fig. 1:**
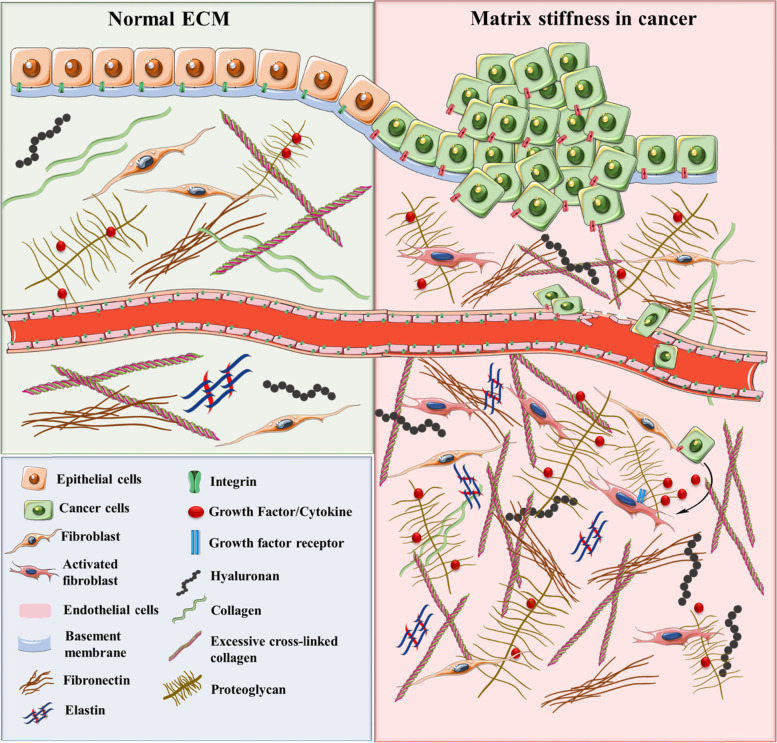
ECM components in normal tissue and in the TME. During stiffening, the ECM is remodelled by the ECM components secreted both by cancer and stromal cells. This, in turn, affects the tissue mechanosignalling and its permeability for different therapeutic agents

It is known that over 700 proteins are implicated in ECM remodelling which have the ability to modify the abundance, concentration, organization and structure of individual ECM components, causing modification in the spatial topology of the matrix which surrounds the cells, and has an effect on cell fate [24]. Although, the ECM represents the interface between tumor niche and normal tissue being able to present both a pro- and anti-tumorigenic role [25]. ECM can also be altered in the context of premetastatic niche formation, generating an environment that would benefit the malignant clone (Fig. 1) [26–28].

## Extracellular matrix proteins

The ECM is composed of various proteins that create specific structures and properties. The ECM is composed of collagen, laminin, proteoglycans and fibronectin, all of which have been shown to be increased in solid tumors (Table 2) [29].

**Table 2:** The role of different type of extracellular matrix proteins in cancer

Component	Detailed	Role in cancer	Reference
Collagen	Type I	Inhibits differentiation and promotes EMT	[30, 31]
	Type III	Associated with drug resistance	[32]
	Type IV	Promotes cancer cell growth	[33]
Proteoglycans	Glypican	Promotes CSC self-renewal	[34]
	Syndecan	Associated with drug resistance	[35]
	Versican	Promotes cancer cell self-renewal	[36]
Non-proteoglycan polysaccharides	Hyaluronan	Affects the acquisition of CSC properties	[37]
Glycoproteins	Laminin	Promotes CSC self-renewal	[38]
	Fibronectin	Promotes EMT	[39]
	Fibrillin	Promotes cancer cell growth	[40]
	Fibulin	Associated with drug resistance	[41]
	Fibrinogen	Promotes cancer cell growth	[42]
	Vitronectin	Sustains CSC self-renewal	[43]
	Nidogen	Promotes EMT	[44]
	Mucin	Associated with growth and metastasis	[45]

Collagen represents the main structural element of the ECM having several characteristics such as mechanical strength, activating cell migration and modifying cell adhesion [46]. Collagen can be classified into several types, including types I, III, IV, VII, XI, XVII having been shown to be involved in epithelial-to-mesenchymal transition (EMT), drug resistance and tumor-initiation [47]. Collagen is a helix glycoprotein formed from three homologous or nonhomologous α chains. These α chain amino acids are formed by glycine-X–Y units, where X and Y might be a proline or hydroxyproline. In this regard, hydroxyproline units found in collagens structure are involved in its stability [48]. From a synthesis standpoint, collagen is released into the ECM where it forms a fibril supramolecular structure. This complex starts in Golgi-to-membrane carriers after procollagen excision. In addition, the stability of collagen is controlled by inter- and intramolecular linkages such as covalent bounds (glycosylation, lysyl oxidation and transglutaminase crosslinks) [49].

Proteoglycans represent an essential component of the ECM require enzymes for a proper production and assembly. The structure of proteoglycans consists of a core protein which is glycosylated with multiple chains of glycosaminoglycans (GAGs). In the Golgi apparatus, the attachment of GAGs occurs where the core protein is translocated. In a wide range of tumors, the proteoglycan differs compared to normal tissues. In prostate cancer, CSPG4 and aggrecan are upregulated, while, the expression level of decorin is downregulated [50]. At the same time, in gastric cancer, the expression level for versican and decorin is elevated alongside an increased glycosaminoglycan content [51]. In squamous cell laryngeal carcinoma, versican and decorin are strongly upregulated, while aggrecan is decreased [52]. Further, another study, has shown that versican is increased in the peritumoral stroma of melanoma [53].

Hyaluronic acid, another key component of the ECM is a glycosaminoglycan which is not conjugated to a protein core and not synthesized in the Golgi apparatus. Synthesis of this protein is assured by a family of three transmembrane glycosyltransferase: hyaluronan synthetase 1–3 (HAS1-3) [54]. In a paper published by Schulz et al*.*, it has been shown that hyaluronan synthetases are responsible for extracellular export of the hyaluronic acid macromolecule through a direct extrusion. Conversely, there are others that assume that hyaluronic acid macromolecules are exported through ABC transporters, including MRP5 or MDR1 [55].

Laminin is a component which belongs to the basement membrane, a structure that appears as continuous and well-delineated. In tumors, the basement membrane and laminin is often distorted [56]. The loss of adherence or the disruption of a basement membrane is a main feature of invasiveness. Interestingly, an increased expression level for laminin and its aberrant distribution is associated with invasiveness and poor prognosis [57].

Hemidesmosomes (HDs) are multiprotein structures involved in the attachment of epithelial cells to the basement membrane, mediating cell-to-matrix adhesion [58]. HDs are mainly constituted from plectin isoform 1a, bullous pemphigoid antigen1 isoform e (BP230, also known as BPAG1), integrin α6β4, bullous pemphigoid antigen 2 (BP180, also known as BPAG2 or type XVII collagen) and CD151 (tetraspanin-24) [59, 60], being responsible for keratinocyte adherence, differentiation, spatial organization of tissue architecture, polarization and survival [61]. In pancreas and colon cancer, it has been shown that hemidesmosomes are disrupted, fact leading to an ECM that is easier to invade by the cancer cells [62, 63].

As it is already known, the composition of ECM in tumors is significantly different compared to that of normal tissues, regarding its composition, architecture, and physical properties. Any alterations that modify the ECM can negatively affect the response to therapy. In this regard, the presence of a dense and rigid ECM engulfs the tumor cells by forming a natural capsid and protecting tumor cells from therapeutics. At the same time, it was observed that, this barrier alters the normal circuit of oxygen, nutrients, and metabolites. The antiapoptotic and drug resistance pathways can be activated via an increased level of hypoxia and metabolic stress, while, an increased tissue stiffness might be linked with chemoresistance mediated via integrin and FAK-signalling [57].

## The ECMs implication in tumorigenesis and cancer progression

The particular architecture and orientation of ECM constituents in the microenvironment of a specific tissue play a critical role in tumor progression, invasion and metastasis [34, 64]. Therefore, ECM provides a large repertoire of growth factors and cytokines, modulating processes such as cell proliferation and adhesion [65]. An important component of the ECM, collagen, is deeply involved and dictates the primary functional properties of the ECM. Any alterations occurring in the deposition or degradation of collagen alter the ECM [66, 67]. These architectural changes cause an increased secretion of collagen I, III and IV and fibronectin, showing that tumor progression involves a continuous interaction between cancer cells and the ECM [68].

As most of the ECM protein is produced by the tumor stroma, and because both the tumor stroma and the ECM have been shown to be involved in various hallmarks of cancer in a myriad of tumors, there have been studies assessing the prognostic impact of ECM in a multitude of cancers. Specifically, these studies have shown that stroma abundance is different between cancers and the quantification of stroma abundance can be of use in predicting the prognosis of these patients [69].

Deregulation of cell shape and alterations occurring in the TME are considered important hallmarks of cancer and can significantly affect cancer cell proliferation. In different types of cancer, collagen, glycoproteins and proteoglycans may present dual role, pro- or anti-tumorigenic properties [70]. Collagen interacts with tumor suppressor genes. Also, collagen changes have also been shown to occur as a result of mutations occurring in proto-oncogenes, thus having an impact in cancer progression, invasion and metastasis [71]. Thus, collagen seems to act similar to a migrator. In an experiment performed by Bonnans et al*.*, it has been shown that a dense amount of collagen I increased the risk of tumor metastasis, this, in turn, being associated with a worse prognosis [72]. It its known that, in normal tissues, collagen fibres are curly with an irregular arrangement, while, in tumor tissues, these are linear and dense with a directional arrangement [73]. It was observed that collagenases are responsible for collagen remodelling in this case. Thus, matrix metalloproteinase (MMP) and lysine oxidase (LOX) family proteins are of main importance, causing the rearrangement of collagen fibers [74, 75]. An increased collagen cross-linking and deposition cause tumor progression through an increased integrin signalling [64, 76]. Added to this, depletion of fibrillar collagen I and III induces malignant progression as well [77, 78]. Collagen cross-linking is regulated by LOX [46]. Cancer cell-secreted LOX increases matrix stiffness and volume. It is known that, by increasing the stiffness of the ECM, integrins are activated and stimulate Rho-generated cytoskeletal tension [79]. The ECM integrins are modulated by crosstalk with different signalling molecules like receptor molecules on the cell surface or present in the cytoplasm as actin-binding proteins and adaptor proteins [80]. COL11A1 was found to be involved in cancer progression through different biological processes such as apoptosis or EMT [81]. In several studies, it was observed that collagen IV-derived canstatin, tumstatin and tetrastatin, and collagen XIX-derived matrikine act through binding to several integrins, including α3β1, α5β1, or αVβ3. As a result, cellular proliferation and invasion are decreased [82, 83].

In some studies, it was found that an increased secretion of collagenases stimulates an abnormal collagen remodelling in breast cancer and hepatocellular carcinoma [84, 85]. An important aspect is that, in cancer cells, the architecture and content of collagen is modified by mutated tumor suppressor genes. In cancer cells, mutated *TP53* affects collagen production [86]. The alteration of collagen in cancer can occur through a multitude of mechanisms, including the alteration of Hsp47, which is linked with collagen deposition [87], but also the overexpression of collagen related genes in cancer cells [88], sustaining resistance to therapy. Added to this, a therapy-resistant ovarian cancer cell line (A2780/A2780cis) was shown to present upregulated *COL1A2, COL12A1, COL21A1, LOX, TGFBI, LAMB1, EFEMP1, GPC3, SDC2, MGP, MMP3*, and *TIMP3* genes. This adds to the evidence that ECM components secreted by cancer cells have an impact in therapy resistance [88].

The collagen production is affected by the presence of mutated *TP53* alongside the activation of JAK2-STAT3 signalling [86]. Also, in another study, it has been shown that Arresten, an antiangiogenic factor being localized in the C-terminal non-collagenous domain of COL4A1, is associated with p53 activation [89]. In this regard, p53 increases the level of collagen prolyl-hydroxylase which, in turn, enhances the production of COL4A1 and the content of Arresten [90]. Another study has shown that loss of *TP53* leads to the activation of JAK-STAT, which, in turn, is able to affect the number of stellate cells in pancreas tumor stroma, the level of periostin, and, subsequently, the structure of collagen [86].

Fibronectin is involved in tumor progression as well as in drug resistance and metastasis with an increased expression level in many solid tumors [91]. An increased deposition of matrix proteins stimulates tumor progression by interfering with cell polarity, cell-to-cell adhesion as well as growth factor-mediated signalling [92]. Several studies have shown the implication of ECM components in tumor progression.

**Fig. 2:**
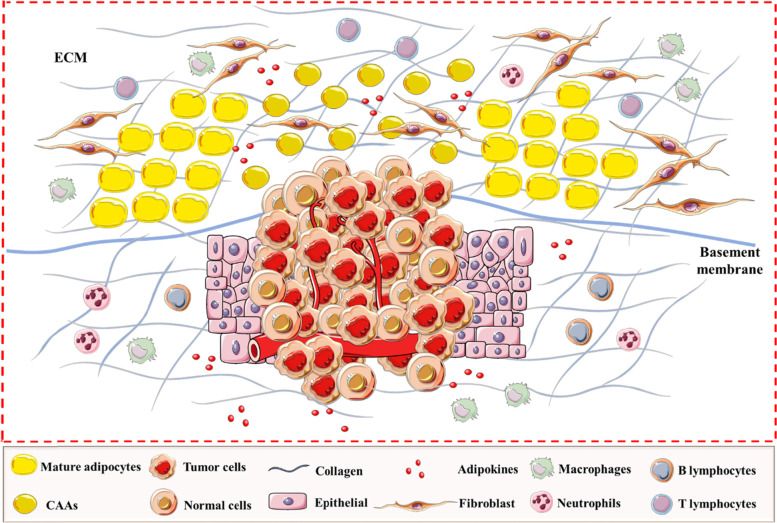
The ECM components implicated in cancer progression and metastasis. Cancer cells are directly exposed to the tumor microenvironment that contains adipocytes when cancer cells break through the basement membrane. Further, adipocytes that surrounded the tumor cells change into CAAs, being characterized by a reduced volume, a gain of an irregular shape with dispersed and small lipid droplets. An important role of these components is involved in sustaining cellular progression and metastasis of cancer cells. Abbreviations: ECM, extracellular matrix, CAAs, cancer-associated adipocytes

ECM stiffness plays a critical role in tumor progression by modulating intrinsic properties of tumor cells. The presence of a rigid ECM can determine intracellular contractions further sustaining cell migration [93]. ECM stiffness induces the activation of TGFβ signalling that mediates EMT in cancer cells. Thus, the interaction between stromal and epithelial cells plays a pivotal role in tumor progression [94] (Fig. 2). In pancreatic cancer cells, the genetic alterations which occur in TGFβ signalling change the activity of STAT3 kinase by increasing the deposition and remodelling the tumor ECM. In this regard, the tumor stiffness is increased and enhances tumor growth [95]. It is already known that TGFβ has a dual role in cancer [96]. In the early stages of cancer, the role of TGFβ is to inhibit tumor cell proliferation, while, in the later stages of cancer, it is to stimulate the malignant progression and metastasis of cancer cells [97]. LTBP3, a protein that regulates TGFβ secretion, supports the invasion and metastasis of the primary tumor and is involved in the formation of the fibrillar ECM network. The SNED1 glycoprotein is a component of the basement membrane, and it has been shown to present a functional role in tumor progression and metastasis [98]. Furthermore, a link between the ECM landscape and the genotypic features of the malignant clone has been observed and was shown to be important in tumor growth [80].

Hyaluronic acid has been shown to be heavily altered in the tumor ECM through the alteration of HA synthases, HA receptors, and HYAL-1 hyaluronidase, which lead not only to an altered expression of hyaluronic acid, but, also, to a different signalling pathway involving hyaluronic acid [99]. Moreover, hyaluronic acid production was proven to have an increased expression level in several types of cancer such as pancreatic carcinoma [100], breast cancer, colorectal cancer, prostate cancer and brain tumors [101], with the increased level of hyaluronic acid being associated with poor prognosis [102]. It has been identified that stromal cells, especially fibroblasts, are the main source of hyaluronic acid in the tumor. Added to this, it was found that hyaluronic acid is implicated in EMT induction and can also act as a migration substrate [103]. In the case of hyaluronic acid, it has been shown that HAPLN1, a protein having the role of linking proteoglycans to hyaluronic acid is overexpressed in metastatic melanoma [104].

## The ECMs’ stromal components are involved in the regulation of epithelial-to-mesenchymal transition (EMT), invasion and metastasis

To provide a comprehensive understanding about critical biological processes, including EMT, metastasis, the key players involved in these processes have to be better understood. The presence of cancer-associated fibroblasts (CAF) in the TME influences ECM composition, further altering EMT and metastasis. Added to this, it has been shown that EMT is characterized by loss of cell adhesion, increased cell motility, inhibition of E-cadherin expression [105], increased metastasis [106] and chemoresistance [107, 108]. In cancer, EMT is linked with the acquisition of a more stem-cell-like character. In a study performed by Fisher et al*.*, it has been shown that tumor cells resistant to cyclophosphamide acquire a mesenchymal character which promotes the expression of drug efflux pumps (ABCC1 and ABCB1) [109].During cancer progression, tumor cells are able to activate immune cells and fibroblasts which secrete different cytokines affecting cancer development and metastasis [110]. More important, it has been found that several cytokines regulate EMT. One of the most important EMT inducers is TGFβ which is secreted by tumor cells, platelets and CAFs. In prostate cancer, it was observed that TGFβ induces TWIST1 and SNAIL2 expression via IKKα and SMAD signalling [111–113]. The activation of the NFkB signalling pathway through TNFα induces the expression of the EMT transcription factors, including SNAIL2, TWIST1 and ZEB1/2 [114, 115]. Also, the activation of NFkB stabilizes SNAIL1 further stimulating cell migration and invasion. In several studies, it has been shown that IL6 increased the expression of TWIST1 and SNAIL1 and promoted EMT in breast and in head and neck cancer [116, 117]. Thus, the inflammatory cytokines from the TME can modulate the expression level and the protein stability of EMT transcription factors leading to the stimulation of EMT and invasion promotion [118].

In addition, tissue stiffness is an aspect that is directly involved in EMT. Increasing evidence shows that an increased stiffness of the surrounding ECM determines EMT by increasing nuclear localization of the transcription factors TWISTs [119], YAP and TAZ [120]. In a study performed by Fattet et al*.*, it was shown that EPHA2/LYN/TWIST1 mechano-transduction pathway, which is associated with collagen fibre alignment, is involved in increasing the ECM stiffness during tumor progression and in stimulating EMT, cell invasion and metastasis [121]. Thus, an increased expression level of vimentin and a decreased level of E-cadherin was observed to be associated with an increased resistance of tumor cells to paclitaxel. In gastric cancer, it has been observed that the interaction occurring between hyaluronic acid and hyaluronan-mediated motility receptor (HMMR) is important in inducing 5-fluorouracil resistance [122]. Hyaluronic acid acts as a ligand for CD44 and plays an important role in EMT, causing an increased in metastasis and invasiveness [123, 124]. By co-culturing tumor cells with fibroblasts, the synthesis of hyaluronic acid is increased. More important, the inhibition of hyaluronic acid reduced the expression of cancer stem cell markers (CD90, CD133, EpCAM) [125].

Another critical aspect is represented by the loss of epithelial polarization in EMT. In a study conducted by Walter et al*.*, it was observed that alterations of the basement membrane and of collagen IV deposition can trigger EMT [126]. The effects of collagen fibres also depend on the collagen type, with collagen IV being important in maintaining an epithelial phenotype, while collagen I being important in stimulating EMT [127]. Also, it was observed that TWIST1 is a direct regulator of collagen alpha1(VI) transcription [128], while, ZEB1 regulates collagen 1 component’s transcription and increases the expression of LOXL2 that is involved in collagen stabilization [129].

Thus, in the pancreatic cancer stellate cells, collagen production is improved through the association between mutated KRAS and EMT regulator SNAIL [49]. Transcription factors can determine an aberrant expression profile of certain transcripts, such as NFkB and STATs which participate in collagen expression and organization. In sarcomatous, COL2A1 is under the transcriptional control of the NFkB subunit p65 [130]. A typical component which belongs to serine/threonine kinase signal transduction, is represented by TGFβ/SMAD signalling involved in collagen modification. Cancer progression is promoted in melanoma cells through the crosstalk between TGFβ and the MAPK/ERK signalling pathway [131]. Added to this, FGFR (fibroblast growth factor receptor) regulates the degradation of COL1, COLII and COLIV by increasing the protein expression of MMP14 in prostate cancer cells [131, 132].

Cartilage oligomeric matrix protein (COMP) also represents a component of the ECM which binds to integrins on the cell surface via the RGD domain (Arg-Gly-Asp) [133]. COMP is a multifunctional protein being expressed in different cell types and tissues [134]. Specifically, it has been shown to promote cancer progression [135, 136], EMT [137] and resistance to therapy [138]. It has been shown in breast cancer epithelial cells, that COMP is significantly elevated being associated with poor survival, invasiveness, and metastasis [136]. In addition, the expression of COMP in breast cancer cells, mediates the interaction between Jagged1 and Notch3, leading to the alteration of the cancer stem cells in vitro and in vivo [139]. In prostate cancer cells, COMP is associated with cancer recurrence. Also, prostate cancer cells which present an increased expression level of COMP are protected against apoptosis and have an enhanced Warburg effect [140]. In colon cancer cells, the knockout of COMP inhibits cell proliferation, reducing 5-Fluorouracil resistance and ultimately induces apoptosis [141].

To create a suitable soil for the colonization of distant sites, metastatic cells are able to create conditions for attachment, survival and growth [26–28]. This microenvironment, is also called pre-metastatic niche [142] by involving ECM remodelling [143]. In the primary ECM, the collagen deposition is increased, but fibronectin, glycoproteins and proteoglycans are the key players in the formation of the pre-metastatic niche [144]. Exosomes secreted by primary tumors, activate stromal cells in the pre-metastatic niche in order to secrete new ECM molecules, or to remodel the ECM, by creating a fibrotic and pro-inflammatory environment. Also, there are several soluble factors which sustain the formation of the pre-metastasis niche. At the pre-metastatic niche, bone marrow-derived cells (BMDCs) contribute to ECM remodelling through integrins and pave the way for the arrival of disseminated tumor cells [145]. Neutrophil elastase and MMP9 cleave laminin, create a specific environment that can activate dormant tumor cells, while, circulating tumor cells reach the distant tissue through the disrupted vasculature [146]. Also, periostin and versican induce EMT and promote metastatic niche formation [147], collagen I, III and IV promote metastasis [148]; TGFβ is released during ECM degradation and fibronectin leads to the production of a pre-metastatic niche in the liver [147].

Several studies made on breast cancer, with Hsp47 having been shown to be an important regulator of ECM genes [87]. Other studies on breast cancer ECM have focused on the direct expression of ECM components by breast cancer cells. In invasive ductal breast carcinoma, the cancer cell expression of the protease inhibitor, CSTA and genes involved in adhesion, *FAT1*, *DST*, and *TMEM45A*, were shown to be involved in cancer invasiveness [149]. Further, collagen prolyl hydroxylases are implicated in breast cancer metastasis [150]. These effects have been shown to also extend in the direction of therapy resistance, with therapy-resistant MCF7 having been shown to present upregulated *MMP1, MMP9, ADAM9* and *TIMP3* genes [151]. More than in breast cancer, other malignancies have been shown to secrete ECM components. Pancreatic circulating tumor cells secrete ECM components, including SPARC, with the knock-out of this gene being associated with reduced invasiveness [152].

## The ECMs’ stromal components are involved in the modulation of the response to therapy

ECM was shown to influence drug response (Table 3). It is known that drug distribution of most compounds in tumor tissues occurs mainly via diffusion. In this regard, solid tumors are characterized by the presence of a condensed ECM that significantly reduces drug transport [57]. Lysyl oxidase isoenzymes, including LOX and LOXL1-4, are responsible for the stabilization of collagen networks. Several studies highlighted the importance of this lysyl oxidase in cancer. In a study performed by Schutze et al*.*, it has been shown that diffusion of doxorubicin in tumor spheroids is hampered by the overexpression of LOX and LOXL2. By using 2-aminopropionitril, the lysyl oxidase activity was inhibited and the effect was reversed [153]. Also, lysyl oxidases have a direct effect on VEGF-A expression through oxidation of PDGFR extracellular domain [154]. In tumor tissues, an increased LOXL2 facilitates endothelial invasion, which is a critical step in neo-angiogenesis, most probably through the increased motility of endothelial cells [155]. An in vivo study performed on murine pancreatic ductal adenocarcinoma (PDAC) models, has demonstrated that hyaluronidases reduce the hyaluronic acid content and increase the uptake of gemcitabine and doxorubicin [156]. The same result was observed in osteosarcoma, where, the uptake of liposomal doxorubicin could be improved with hyaluronidase treatment [157].

**Table 3:** Implication of ECM in response to therapy for solid tumors

Cancer type	Study model	Treatment	Effect on ECM component	Reference
Lewis lung carcinoma	In vitro—3D models	Doxorubicin	Diffusion of doxorubicin in tumor spheroids is hampered by the overexpression of LOX and LOXL2	[153]
Fibrosarcoma				
Breast carcinoma				
Pancreatic ductal adenocarcinoma	In vivo—murine model	Doxorubicin	Hyaluronidases reduce the hyaluronic acid content and increase the uptake of gemcitabine and doxorubicin	[156]
Osteosarcoma	In vivo—murine model	Liposomal doxorubicin	Uptake of liposomal doxorubicin is improved by hyaluronidase treatment	[157]
Small cell lung cancer	In vitro—cell lines	Etoposide	Expression of survival signals through ILK/Akt/NFkB determines the interaction between fibronectin and integrin β1, protecting against etoposide	[158]
Ovarian carcinoma	In vitro—cell lines	Docetaxel	Silencing FAK in ovarian carcinoma sensitizes cells to docetaxel	[159]
Colorectal carcinoma	In vitro—cell lines	5-fluorouracil	Silencing FAK in colorectal carcinoma sensitizes the cells to 5-fluorouracil	[160]

**Fig. 3:**
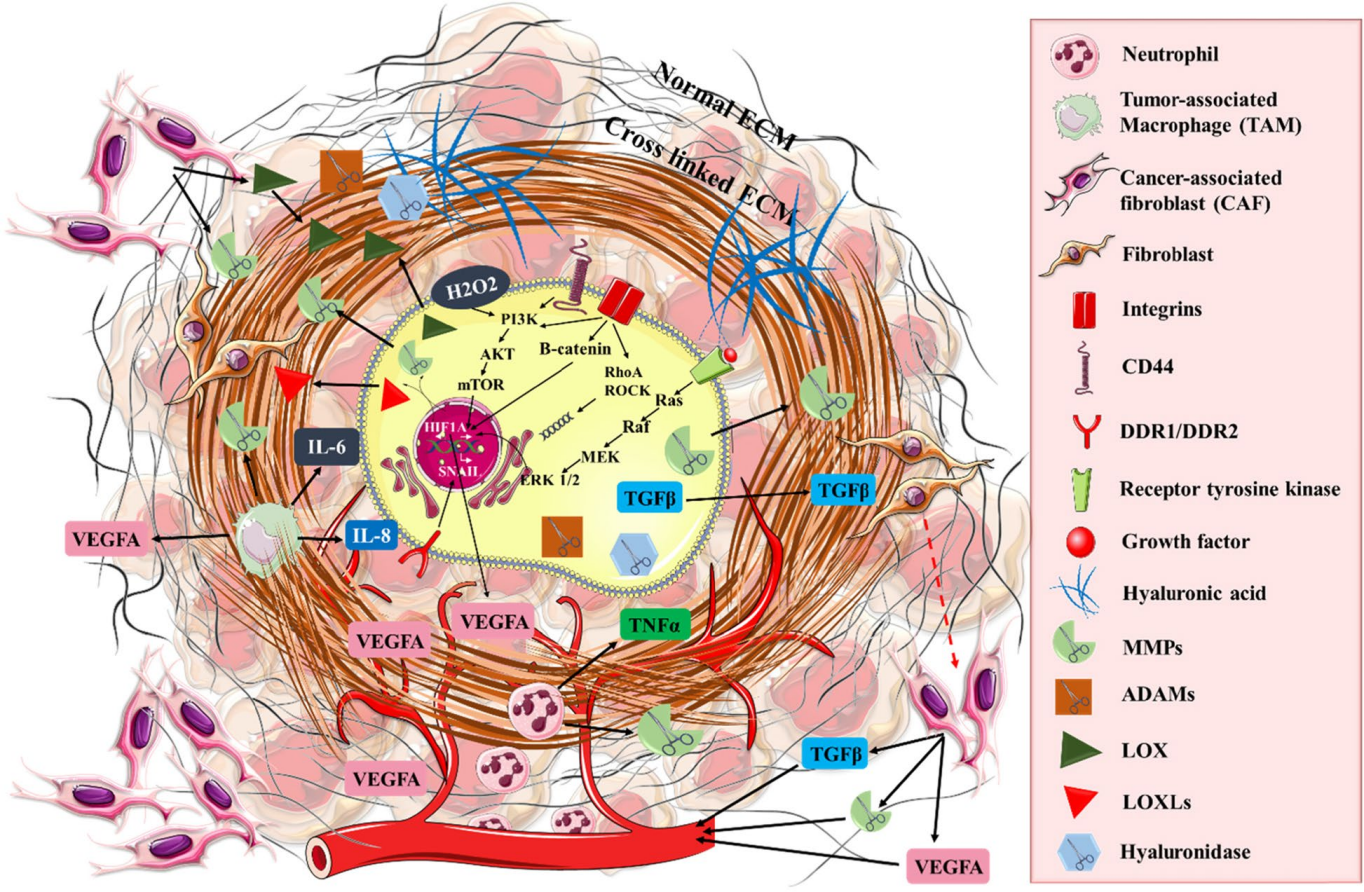
The role of ECM in the TME. There is a bidirectional communication between the malignant clone and the TME associated cells, which ultimately leads them to increase their secretion of proteases, crosslinkers and soluble cytokines/chemokines. The altered ECM resulting from these changes in cell secretion subsequently has an impact in affecting the malignant clone and the TME. Specifically, it can be observed how the malignant clone can alter the properties of surrounding fibroblasts, transforming them in cancer associated fibroblasts, which, subsequently secrete VEGFA, with important proangiogenic properties. Another important cell that communicates with the malignant clone and has an influence on the ECM is represented by the tumor associated macrophage, which secretes key inflammatory cytokines which indirectly alter the ECM. Added to this, the malignant clone itself is able to secrete matrix metalloproteinases, which affect the structure of the surrounding ECM, making it more appropriate for tumor development

Cells present a direct interaction with the TME through various cell surface receptors (Fig. 3). These receptors inform the cells of any alterations occurring in the TME regarding ECM composition and stiffness, as well as responses that alter the cells sensitivity to drugs. Many studies showed the implication of these cell surface receptors. In small-cell lung cancer cells, the interaction between ECM and integrin β1 prevents G2/M arrest in response to chemotherapy or radiation by increasing the expression of p21 and p27 and downregulating the expression of cyclin A, B and E [158]. Another study showed that an upregulated expression of survival signals through ILK/Akt/NFkB determines the interaction between fibronectin and integrin β1 and protection against etoposide [158]. FAK signals are also involved in activating pro-survival pathways such as MAPK and AKT, which confer resistance to mTOR inhibition [161]. By silencing FAK in ovarian carcinoma it was observed that cells become sensitive to docetaxel [159], while, in colorectal carcinoma, cells acquired a sensitive phenotype to 5-fluorouracil [160].

In tumors, the stiffened matrix is mainly involved in drug resistance by reducing it and presenting a poor therapeutic efficacy of chemo- and immune therapies [162]. In the first step, a physical barrier for drug infiltration is formed by the stiffened matrix. To confirm this aspect, it was shown that decreasing HA deposited in tumor tissue was associated with systemic accumulation of chemotherapy in patients with colorectal cancer [163]. Secondly, a stiff matrix has micro blood vessels causing difficulties for the drugs to reach the central core of the tumor. Third, hypoxia is induced by ECM stiffness [164]. Fourth, in this context, tumor cells are forced to transform to cancer stem cells (CSCs) with the ability to proliferate in a hypoxic environment. In this regard, several studies, revealed that CSCs are more resistant to chemotherapy [165, 166]. It was shown that β1 integrins have an elevated expression profile in several types of cancer, such as colorectal [167] and lung cancer [168]. More important, an increased profile of β1 integrins contribute to tumor cell survival in prostate, pancreatic, melanoma, glioblastoma and colorectal carcinoma after radiation [168–170].

Also, the response of matrix stiffness to drug resistance might be linked to the drug and cell type. There are some studies performed on cell lines which tried to highlight the importance of matrix stiffness. In one study Medina et al*.* showed that, using a matrix to mimic the stiffness of their host tumor, mammary cancer cells acquired an increased drug resistance potential [171]. As a major point of view, by changing the matrix stiffness, drug resistance could be modulated in an advantageous way. A study performed on breast cancer cells, revealed that a stiff 2D matrix induces resistance to sorafenib and lapatinib, while, drug resistance mechanism is reduced in a stiff 3D matrix [172]. To provide a better understanding regarding in vitro drug resistance, studies might focus on 3D culture models using different matrix stiffness for developing new therapeutic strategies [173]

A wide range of studies revealed that collagen might be used as a prognostic factor correlated with cancer invasion and differentiation, lymph node metastasis and clinical stages in cancer patients. It was shown that the content of COLI and COLIV is associated with proliferation, potentially representing future biomarkers [174, 175]. In addition, the expression level of COLI and COLIV can offer information about tumor angiogenesis and progression of glioblastoma [176]. In several types of cancer, including breast cancer, lung adenocarcinoma, squamous cell carcinoma, cervical cancer and neck squamous cell carcinoma, it was observed that hypomethylation of the COL17A1 promoter is associated with an advanced stage and sustained invasion of tumor cells [177].

Neo-epitope biomarkers showed promising results in characterizing localized pathological protein turnover in different diseases. Thus, an important biomarker for bone turnover is represented by carboxy-terminal collagen crosslinked (CTX-1) which is cleaved by cathepsin K[178]. The altered activity of MMP, ADAMTS, calpain and caspase proteases are involved in protein turnover changes in several pathologies. The changes of MMP-generated collagen VI are linked to muscle regrowth following immobilization [179]. The collagen I fragments which are generated by MMP2, 9, and 13 (C1M) [180] or the MMP9 proteolytically showed neo-epitope biomarkers of type III collagen (C3M) used as muscle protein turnover [181]. In a recent study, Groen et al. developed a neo-epitope biomarker for osteoarthritis, based on MMP1 and 13 generated fragment of type II collagen degradation (T2CM) [182]. A well-known marker, type X collagen (Col10) was developed as a new neo-epitope biomarker for radiographic knee osteoarthritis [183]. GPDPLQ_1237_ was developed as a promising biomarker for cartilage degradation in vitro [184] and PRO-C3 collagen for fibrogenesis [185].

Additionally, collagen plays an essential role in therapy resistance. In esophageal carcinoma, it was shown that an elevated level of collagen is correlated with chemotherapy resistance [186]. The increased content of collagen is associated with an elevated accumulation of hyaluronan, causing resistance to doxorubicin in pancreatic cancer [187]. In several studies, it was observed that COLI induces resistance to cisplatin and mitoxantrone in ER-positive cancer cells, by activating β1 integrin followed by the FAK/PI3K/AKT pathway, in triple negative cancer cells by MAPK pathway and in ovarian cancer through the co-expression of LOX with COL1A2 [188, 189]. The upregulation of COLI caused paclitaxel resistance in ovarian carcinoma [190], as well as COL11A1 by mediating the transcriptional activation of NFkB to increase the expression of Twist family [191]. Anoikis resistance is caused by an upregulated COLVI profile, affecting the response of colorectal cancer to adjuvant chemotherapy and of salivary gland cancer to radiotherapy [192, 193]. The response of cancer cells to antiangiogenic therapy is strongly associate with COLIV expression. Thus, the resistance to VEGF therapy through TGFβ signalling, is causing by the reduction of collagen to PDAC cell surface receptors [194]. In addition, collagen can be used in clinical applications as a therapy resistance biomarker, as a predictor of prognostic and recurrence, as a drug carrier and as a therapy target [49].

### Cancer-associated fibroblasts

CAFs are one of the most dominant cell types in the TME and are found in all solid tumors [195]. CAFs mediate the onset of a pro-invasive TME through multiple epigenetic, transcriptomic or genetic alterations [196]. CAFs are different between each type of tumor, influence angiogenesis, tumour mechanics, drug access and therapy responses [197]. In this regard, normal fibroblasts, epithelial cells, endothelial cells [198], adipose tissue-mesenchymal stem cells, stellate cells, bone marrow-derived mesenchymal stem cells, as well as resident quiescent fibroblasts were found to be the cells of origin of CAFs [199, 200]. According to their origin, CAFs exhibit several differences in morphology, cell-to-cell interaction and expression profile [201]. CAFs are able to secrete proteases which increase their ability to migrate and remodel the ECM, as well as inflammatory molecules and growth factors [202].

In breast cancer, it has been observed that the connection between tumor cells and fibroblasts promotes the CAFs phonotype via Notch signalling [197, 203]. Several inflammatory modulators are able to activate CAFs through IL1 acting via NFkB and IL6 acting on STAT factors [197, 204]. Also, the contractile cytoskeleton and alterations in histone acetylation stimulate CAF activation through JAK-STAT signalling [196]. Physical changes occurring in the ECM also play an important role in CAFs activation [205, 206]. In this regard, the association between CTGF and CYR61, and contractile cytoskeleton have the role to increase tissue stiffness causing SRF-dependent and YAP1-dependent transcriptional programmes, which further lock CAFs into a self-sustaining positive-feedback loop [207]. The presence of HSP1 is also required for CAFs activation [208]. Alongside physical changes, physiological and genomic stress are also important for changes occurring in fibroblasts. In some cases, the production of IL6 and TGFβ caused a non-proliferative state of fibroblasts called senescence [209]. The senescent fibroblasts are a minor component of the TME [210] and their absence has substantial consequences for disease relapse [209]. In CAFs it was observed that YAP establishes and maintains tumor-promoting functions, including invasion, angiogenesis, and stromal sclerosis. An increased level of YAP is associated with positive feedback between mechanical conduction and CAF-driven matrix hardening [207]. Also, YAP is activated in CAFs in response to mechanical stress, ECM stiffness and alterations of the actin cytoskeleton [211].

Following therapy, cancer cell activities are affected through multiple signalling pathways, which alter TME components. In this regard, CAFs secrete various cytokines that activate signalling cascades to hinder the elimination of the cancer cells. Thus, CAFs contribute to drug resistance through several mechanisms, via PAI-1 which suppresses caspase-3 activation and activates Erk/Akt signalling [212]. Through the production of IL6, CAFs induce the expression of CXCR7 through the STAT3/NFkB pathway in tumor cells, which is responsible for drug resistance [213]. The co-culture of CAFs in conditioned media from gastric cancer cells showed an increased level of phosphorylated AKT, PI3K, p65, lkb and ABCB1, together with the activation of NFkB that increased the resistance to cisplatin in cancer cells [214]. Performing a cytokine antibody array on a multidrug-resistant breast cancer cell line, MCF-7/R, it was observed that IL6 and IL8 exhibited a significantly increased level of these cytokines, suggesting their implication in drug resistance [215]. Under hypoxic conditions, it was observed that CAFs secrete an increased level of TGFβ, causing stem cell-like properties and increased chemoresistance in colon cancer cell lines [216]. In order to counteract drug resistance mechanisms, CAFs are reprogramed to a quiescent phenotype by inhibiting NFkB signalling. In a study performed on ovarian cancer, it was demonstrated that inhibition of NFkB reversed the typical CAFs phenotype leading to an improved response to cisplatin in tumor cells [217]. Added to this, NFkB inhibition reduced the deposition of collagen in the TME after cisplatin therapy. It has been shown that WNT16B is regulated by NFkB, after the activation of DNA damage mechanisms, which might be induced by TNFα and radiotherapy. This mechanism targets the canonical Wnt pathway leading to drug resistance [218]. In pancreatic cancer, it was observed that CAFs protect cancer cells from cell death induced by gemcitabine in an NFkB-dependent manner. The apoptosis process was regulated by IL1β and IRAK4 where, by knocking down IRAK4 or inhibiting IL1β, drug resistance is increased, but fibrosis is decreased [219]. Due to the fact that CAFs express vitamin D receptor, using calcipotriol, CAFs were converted to stellate cells causing an increased concentration of gemcitabine in the tumor site improving the response to therapy [220]. In breast cancer, it was observed that collagen density decreased the number of CD8 + tumor-infiltrating T cells (TILs) and modulated T-cell cytotoxic activity [221]. In the same malignancy, using an in vivo model, it has been found that CAFs reprogramming is mediated through hedgehog ligand, leading to stemness and drug resistance through the expression of FGF5 and production of fibrillar collagen [222]. Smoothened inhibitors (SMOi) can reduce the expression of cancer stem cell markers and sensitize cells to taxanes by reducing metastasis, thus improving the survival rate [222]. In addition, it has been observed that CAFs regulate EMT and promote chemoresistance by secreting IL6 and HGF [223]. In non-small cell lung cancer (NSCLC), CAFs increase the expression of TGFβ1 by secreting IL6, further increasing the resistance to cisplatin [224]. A study performed by Ying et al*.*, showed that an increased expression of HGF secreted by CAFs enhanced the activation of PI3K/AKT pathway and GRP78, causing resistance in NSCLC to paclitaxel. Also, CAFs induce EMT in NSCLC causing resistance to chemotherapy [225].

Another important factor that sustains drug resistance is the maintenance of CSCs. In colorectal cancer, drug resistance is promoted in cancer cells through IL17A secreted from CAFs [226]. It was found that Wnt signalling is critical for establishing drug resistance during cancer progression, but exosomal Wnt from CAFs are able to induce a drug resistant phenotype to cancer cells [227]. The microvesicles secreted from CAFs transfer miR-221 to breast cancer cells that activate an ER^low^/Notch^high^ feed-forward loop responsible for CD133^high^ CSCs generation. Drug resistance was observed to occur when using patient-derived xenograft (PDX) models [228]. The presence of non-coding RNA H19 from CAFs promotes cancer stemness and drug resistance in colorectal cancer through the activation of the β-catenin pathway [229]. In colorectal cancer, it was shown that hypoxic TME stimulates the stemness and resistance of CSCs to chemotherapy. Thus, a hypoxic TME can induce the aggregation of HIF-1α and TGFβ2 secreted from CAFs, as well as promoting the expression of GLI2 in CSCs, which are responsible of enhancing stemness and therapy resistance in CSCs. An increased expression of HIF-1α/TGFβ2/GLI2 was shown to be related to the risk of recurrence in patients undergoing chemotherapy [216]. Specifically, it was found that the TME and CAFs are the major concern for drug resistance development in tumors. In colorectal cancer, cetuximab can induce CAFs to secrete a large amount of EGF which mediates MAPK signal transduction, ultimately leading to cetuximab resistance [230]. Patients diagnosed with pancreatic cancer which exhibited an increased expression on miR-21 are more likely to be resistant to gemcitabine therapy. An elevated expression of miR-21 promotes the activation of CAFs leading to an increased resistance to gemcitabine therapy, while a downregulation of miR-21 expression is associated with an improved efficacy of gemcitabine therapy [231].

### Cancer-associated adipocytes

Cancer-associated adipocytes (CAA) are important players of the TME being involved in tumor progression, angiogenesis, invasion, metastasis, and drug resistance through the secretion of adipokines such as leptin, adiponectin, CCL2, CCL5 and IL6 [232, 233]. An increased expression of leptin which was found in CAAs can stimulate cell proliferation and angiogenesis by increasing the expression of cyclin D1 and VEGF/VEGFR, upregulation of lysyl hydroxylase enzyme, as well as the activation of several signalling pathways such as ER, JAK/STAT and PI3K/AKT [234, 235]. In addition, it was observed that adiponectin is downregulated in CAAs. It is known that adiponectin has an anti-tumorigenic role by suppressing several biological processes, including growth, and the invasion process of tumor cells [232]. Another study showed that leptin has the ability to activate the focal adhesion kinase, increase metalloproteinase (MMP2 and MMP9) secretion needed for the ECM remodelling and cell migration, and diminish cell-to-cell adhesion [236]. Also, IL6 is secreted by CAAs and was observed to promote cancer progression when adipocytes were co-cultured with breast cancer cells [237]. Interestingly, some have shown that the increased expression of MMP2 and MMP9 might inhibit breast cancer invasion and metastasis through emodin by down-regulating CCL5 in adipocytes [225]. Collagen IV is essential in defining the ECM environment for normal and tumor cells and contributes significantly to tumor growth [238]. In a study performed by Park et al*.*, the effect of collagen IV-derived endotrophin was investigated, which showed a strong effect on growth, angiogenesis, and tissue fibrosis in breast cancer. Also, endotrophin promotes EMT by enhancing TGFβ signalling, causing aggressive and high metastatic breast cancer growth [239]. Therefore, the production of IL6 regulates cell survival, immune suppression and drug resistance through the activation of JAK/STAT [240].

Another study has shown that the protein level of MMP3 in lung tumors is higher in obese patients compared to non-obese patients. Thus, adipocyte-derived exosomes increase the MMP3 expression in lung cancer cells. At the same time, in lung cancer cells, MMP3 increases the activity of MMP9, causing the invasion of cancer cells in vitro and in vivo [241]. An increased expression profile of miR-21 in CAAs was also observed. MiR-21 is transferred via exosomes from CAAs to cancer cells. In this aspect, exosomal miR-21 inhibits apoptosis in ovarian cancer cells and promotes drug resistance by combining with APAF1 [242].

### Endothelial cells

Endothelial cells interact with the ECM and offer the support for angiogenesis. The role of angiogenesis is to assure nutrients and to remove waste, both required for tumor growth. The key activator of endothelial cells is VEGF. This molecule stimulates endothelial cell migration into a normal tissue, and initiates several processes such as cell division and self-organization into vessels [243]. Meanwhile, in tumor tissues, these cells are leaky and are able to form a denser network [244]. At the tumor site, it is known that the ECM is altered, suggesting that endothelial cells present an altered sprouting angiogenesis and cell-to-cell adhesion formation [245]. By controlling the physiological properties of the ECM such as density and mechanics, it is possible to modulate the vascular network [246]. When subjected to an ECM with increased stiffness, tumor-associated endothelial cells generate vessels in an irregular manner, fact caused by their inability to orient themselves [247]. Therefore, the elasticity property of the ECM modulates cell proliferation and angiogenesis by controlling the level of transcription factor that regulates the expression of *VEGFR2* [248].

Glycocalyx (GCX) is represented by a mixture of carbohydrates attached to proteins, which have the ability to regulate the access of cells and bioactive molecules to the endothelium in the blood stream. Therefore, CGX modulates essential processes such as microvascular homeostasis and pathogenesis [249]. Recently, the attention was focused on the endothelial GCX (eGCX) which might serve as a key factor in disease progression [249]. The eGCX marks the luminal surface of the vascular endothelium and interacts with plasma proteins and lipids. Being an important component of the vascular wall, the eGCX modulates vascular homeostasis and hydraulic conductivity [250], maintains the endothelial barrier function by acting as a molecular filter for the endothelium. The eGCX modulates the molecular binding to the endothelial cells surface, according to the high density of anionic charges on its GAGs side chains. Sulfate residues carry the negative charge of eGCX, favouring the docking of positively charged molecules [251]. Specifically, because endothelial GCX can influence endothelial cells stability, it can also be involved in angiogenesis and influence therapy response [249, 252].

### The effects of ECM on immunotherapy

Immunotherapy started to gain attention in the oncology field by using several therapeutic approaches based on patient’s immune system against cancer. In the present, there are several clinical trials based on the ECM in order to improve the response to therapy, and not only related to the functioning of immune cells (Table S1). Checkpoint inhibitors are widely used in the field of oncology, with good results. Nonetheless, tumor cells can express checkpoint inhibitors, like PD-L1, and inhibit T-cell activity. The most well-characterized interaction in cancer immunotherapy is between CD80/CD86-CTLA4 and PDL1-PD1 [253, 254]. An increased number of evidences showed that several types of cancer, including melanoma and NSCLC, present a high rate of response to immunotherapy and checkpoint inhibitors [255, 256]. In some cancer patients, the application of immune therapy is impeded due to the low T-cell infiltrate. Added to this, the infiltration rate is also blocked by the ECM which acts as a protective shield. Thus, an increased density of the tumor ECM is responsible for the distribution of immunomodulatory drugs, as well as the infiltration of immune cells into the tumor [257]. A poor diffusion in tumor ECM causes increased hypoxia and metabolic stress, which determines an upregulation of immunosuppressive factors, including CCL18, CCL22, IL10, TGFβ, and prostaglandin E2 and VEGFA [257]. It is known that TGFβ in the TME acts as a suppressor of infiltrating CD8^+^ cytotoxic lymphocytes (CTL) and natural killer (NK) cells [258]. The aim of VEGFA in the TME is to recruit regulatory T cells (Tregs) that express a VEGFA co-receptor, called NRP1, further suppressing the activation of CTLs [259]. In a study performed by Salmon et al*.* it was observed that, in lung cancer, the migration and distribution of T cells is controlled by aligned collagen fibers that surround the tumor site, as well as perivascular regions in the tumor stroma. Thus, T cells are trapped in the stroma area without being able to reach the cells targeted for destruction. Nonetheless, when using collagenase treatment, an increased T cell infiltration was observed [260]. Another study performed on pancreatic ductal adenocarcinomas, showed that the dense architecture formed by hyaluronan acts in a similar manner as collagen, by preventing infiltration of effector immune cells [156]. A hypoxic TME, caused by a dense architecture of ECM, induces an increased expression of VEGF. The CTL infiltration is reduced and cannot attach on the cell surface proteins of endothelial cells, due to the fact that endothelial cells reduce the expression of cell surface glycoproteins in these conditions, including selectins and adhesion molecules (VCAM1, ICAM1, ICAM2), which are needed for T cell homing [261, 262]. Another study showed that VEGFA promotes immune escape by inducing Treg and PD1 expression on CD8^+^T cells expressing VEGFR2 [263]. Added to this, CAFs release various chemokines which modulate the immune landscape within the TME [264].

The ECM components are able to control the differentiation process of the Treg cells. For example, osteopontin inhibit Treg differentiation through CD44, hyaluronan stimulates the production of IL10, which, is in turn responsible of Treg stimulation [265]. Moreover, it has been shown that the ECM rigidity strongly affects the activation, proliferation, and differentiation of T cells [266]. In a study performed on pancreatic cancer, it has been shown that, by reducing the tumor tissue stiffness and total collagen, myofibroblast depletion caused an extensive remodelling of the tumor ECM. Thus, myofibroblast depletion is linked with Teff/ Treg reduction, causing suppression of immune surveillance. In myofibroblasts depleted mouse tumors, it was observed that suppressed immune surveillance is associated with the stimulation of Tregs [77].

Immune and inflammatory responses can be modulated by metalloproteinases through degradation of the ECM. It is known that metalloproteinases are responsible for the release of immunoactive factors from the cells surface. For example, MMP9, ADAM10 and ADAM17 are involved in MICA shedding from tumor cells [267]. In several malignancies, including prostate and breast cancer, as well as osteosarcoma, an elevated expression profile of metalloproteinase allows the tumor cells to escape immune surveillance [268, 269]. Degradation of collagen I by MMP8 and MMP9 produces the acetylated proline-glycine-proline tripeptide that mimics CXCL8 thus recruiting neutrophils [270]. In chronically inflamed lung, it was found that peptides resulting from elastin digestion by MMP12 act as chemoattractants for monocytes [271]. Fibronectin, tenascin and hyaluronan bind TLR2/4 promoting an inflammatory response [57].

In the TME, the most frequent immune cells are tumor-associated macrophages (TAMs), which mediate adaptative immune responses in cancer. The ECM components are involved in TAM polarization. Thus, it was observed that collagen and hyaluronic acid cause M2 polarization, while fibronectin skews macrophages to an M1 polarization, enhancing the cytotoxic activity of macrophages towards tumor cells [57]. Also, it was found that NK cells are supressed by transmembrane collagens such as Collagen XVII [272].

Although there have been various approaches to targeting ECM in cancer, there still has not been the breakthrough that scientists and clinicians have been looking for. There is no one correct answer for this fact, but there are some hypotheses that have been launched in the community. Firstly, targeting ECM alone might lead to the development of compensatory mechanisms that would offer the tumor another way to thrive [57]. Secondly, because the ECM is composed of a myriad of molecules and because it differs between different tissues, it is rather difficult to choose the optimal target [57].

## Conclusions

The ECM is strongly involved in determining the TME architecture and the different hallmarks of cancer acquired by the malignant cells. Due to the fact that the ECM is a complex mixture of different macromolecules further studies are still needed to better investigate the effect of all ECM components and the interactions between them and the malignant clone. Further, other components are also present in the TME besides the malignant clone and the ECM, represented by the associated cells. These are not only able to influence the malignant clone, but to also have a bidirectional communication with the ECM. Dysregulation of ECM components sustain the design of a favourable environmental niche for tumor cells. Beside critical biological processes such as tumor growth and proliferation, invasion and migration, apoptosis, and metastasis, TME is a key player in the drug resistance of cancer cells. To provide a comprehensive understanding regarding TME modulation of drug resistance activation in different cancer types, advanced researches are needed to identify critical signalling pathways. The ECM components are critical for tumor sustainability and progression. Thus, the better understanding of these can not only lead to more insights into the biology of tumors but can also lead to more therapeutic options for various cancers.

## Supplementary Information

**Additional file 1: Table S1. **Clinical trials of cancer therapies that target ECM and molecules linked with ECM.

## Data Availability

Not applicable.
