# Supplementary material for: The extracellular matrix alteration, implication in modulation of drug resistance mechanism: friends or foes?
Source: J Exp Clin Cancer Res. 2022 Sep 16;41:276. doi: 10.1186/s13046-022-02484-1 (PMC9479349; doi:10.1186/s13046-022-02484-1)
Supplement: Supplementary file 1 — Additional file 1: Table S1. Clinical trials of cancer therapies that target ECM and molecules linked with ECM. [file 13046_2022_2484_MOESM1_ESM.docx]

**Table S1.** Clinical trials of cancer therapies that target ECM and molecules linked with ECM.

| **Therapeutic compound** | **Target** | **Disease** | **Phase** | **Status** | **Hypothesis** | **Observation** | **Reference** |
| --- | --- | --- | --- | --- | --- | --- | --- |
| nab-Paclitaxel + carboplatin | Gp60 | Breast Cancer | 3 | Not yet recruiting | With albumin nanoparticles as a carrier, nab-P increases the concentration of extra-tumor drugs by passing through the albumin receptor (Gp60) transmembrane pathway and the secreted protein acidic and rich in cysteine (SPARC) approach that binds to the extracellular matrix of the tumor. | Treatment of Triple-negative Breast Cancer with Albumin-bound Paclitaxel as Neoadjuvant Therapy: a Prospective RCT | NCT04137653 |
| COL-3 | MMP2/9 | Lymphoma; Melanoma Neoplasm Metastasis; Renal Cell Carcinoma | 1 | Completed | It is hypothesized than an imbalance between MMPs and MMP inhibitors allows the destruction of the extracellular matrix and enhances the ability of the tumor cells to grow and metastasize. By inhibiting MMPs, it is thought that angiogenesis and metastasis can be inhibited. | A Phase I Study of Oral COL-3 (NSC-683551), a Matrix Metalloproteinase Inhibitor, in Patients with Refractory Metastatic Cancer | NCT00001683 |
| SST0001 (Roneparstat) | Heparanase | Multiple Myeloma | 1 | Completed | The heparanase enzyme is a promising target for development of new anticancer drugs. HS and the structurally related heparin are present in most animal species. As an analogue of the natural substrate of heparanase HS, heparin is considered to be a potent inhibitor of heparanase. | SST0001 (Roneparstat) in Advanced Multiple Myeloma | NCT01764880 |
| Simtuzumab (GS-6624) | LOXL2 | MPN | 2 | Completed | Simtuzumab reduces the last step in fibrosis formation. | Efficacy and Safety of Simtuzumab in Adults with Primary, Post Polycythemia Vera or Post Essential Thrombocythemia Myelofibrosis | NCT01369498 |
|  |  | Metastatic Pancreatic Adenocarcinoma | 2 | Completed | LOXL2 plays a role in tumor progression. | A Study to Evaluate the Efficacy and Safety of Simtuzumab Combined with Gemcitabine for Metastatic Pancreatic Adenocarcinoma | NCT01472198 |
|  |  | Colorectal Cancer | 2 | Terminated |  | Efficacy and Safety of Simtuzumab (SIM) With FOLFIRI as Second Line Treatment in Colorectal Adenocarcinoma | NCT01479465 |
| Andecaliximab (GS-5745) | MMP9 | Gastric Adenocarcinoma; Gastroesophageal Junction Adenocarcinoma | 2 | Completed | Andecaliximab inhibits MMP9, thus reducing invasion and metastasis. | Study to Evaluate the Efficacy and Safety of Andecaliximab Combined with Nivolumab Versus Nivolumab Alone in Adults with Unresectable or Recurrent Gastric or Gastroesophageal Junction Adenocarcinoma | NCT02864381 |
|  |  | Gastric Adenocarcinoma | 1 | Terminated |  | Study to Evaluate the Safety and Tolerability of Andecaliximab as Monotherapy and in Combination with Anti-Cancer Agents in Japanese Participants with Gastric or Gastroesophageal Junction Adenocarcinoma | NCT02862535 |
|  |  | Gastric Adenocarcinoma | 3 | Completed |  | Andecaliximab With mFOLFOX6 as First Line Treatment for Advanced Gastric or Gastroesophageal Junction Adenocarcinoma (GAMMA-1) | NCT02545504 |
|  |  | Pancreatic Cancer; Non-small Cell Lung Cancer; Esophagogastric Cancer; Colorectal Cancer; Breast Cancer | 1 | Completed |  | Study to Evaluate the Safety and Tolerability of Andecaliximab as Monotherapy and in Combination with Chemotherapy in Participants with Advanced Solid Tumors | NCT01803282 |
|  |  | Glioblastoma | 1 | Not yet recruiting |  | Phase I Study of Monoclonal Antibody (GS) 5745, a Matrix Metalloproteinase 9 (MMP9) Mab Inhibitor, in Combination with Bevacizumab in Patients with Recurrent Glioblastoma (MARELLE01) | NCT03631836 |
| PEGPH20 | HA | Pancreatic Adenocarcinoma Non-resectable | 2 | Terminated | PEGPH20 degrades HA and decreases the vascular compression determined by the tumor. | PEGPH20 Plus Gemcitabine with Radiotherapy in Patients with Localized, Unresectable Pancreatic Cancer (HALO-IST) | NCT02910882 |
|  |  | Pancreatic Cancer | Not Applicable | Completed |  | Two Stage Study of Single Dose PEGPH20 And Cetuximab in Patients with Pancreatic Adenocarcinoma Prior To Surgical Resection | NCT02241187 |
|  |  | Non-small Cell Lung Cancer; Gastric Cancer | 1 | Completed |  | A Study of PEGylated Recombinant Human Hyaluronidase (PEGPH20) With Pembrolizumab in Participants with Selected Hyaluronan High Solid Tumors | NCT02563548 |
|  |  | Non-small Cell Lung Cancer | 1 | Terminated |  | A Phase 1b Study of PEGylated Recombinant Human Hyaluronidase (PEGPH20) Combined with Docetaxel in Subjects with Recurrent Previously Treated Locally Advanced or Metastatic NSCLC (PRIMAL) | NCT02346370 |
|  |  | Pancreatic Cancer | 1 | Terminated |  | A Trial of PEGPH20 in Combination with Avelumab in Chemotherapy Resistant Pancreatic Cancer | NCT03481920 |
